# Transthoracic echocardiography reference values in juvenile and adult 129/Sv mice

**DOI:** 10.1186/1476-7120-11-12

**Published:** 2013-05-01

**Authors:** Maurícia Vinhas, Ana Carolina Araújo, Sónia Ribeiro, Luís Brás Rosário, José António Belo

**Affiliations:** 1 Regenerative Medicine Program, Department of Biomedical Sciences and Medicine (DCBM), University of Algarve (UALG), Campus of Gambelas, Edf.8, 8005-139, Faro, Portugal; 2 Institute for Biotechnology and Bioengineering, Center for Molecular and Structural Biomedicine (CBME), University of Algarve, Campus of Gambelas, Edf.8, 8005-139, Faro, Portugal; 3 Santa Maria Hospital, Cardiology Service, Lisbon Academic Medical Centre (CCUL), Lisbon, Portugal

**Keywords:** Echocardiography, Juvenile, Adult, 129/Sv mouse, Reference values, Doppler

## Abstract

**Background:**

In the recent years, the use of Doppler-echocardiography has become a standard non-invasive technique in the analysis of cardiac malformations in genetically modified mice. Therefore, normal values have to be established for the most commonly used inbred strains in whose genetic background those mutations are generated. Here we provide reference values for transthoracic echocardiography measurements in juvenile (3 weeks) and adult (8 weeks) 129/Sv mice.

**Methods:**

Echocardiographic measurements were performed using B-mode, M-mode and Doppler-mode in 15 juvenile (3 weeks) and 15 adult (8 weeks) mice, during isoflurane anesthesia. M-mode measurements variability of left ventricle (LV) was determined.

**Results:**

Several echocardiographic measurements significantly differ between juvenile and adult mice. Most of these measurements are related with cardiac dimensions. All B-mode measurements were different between juveniles and adults (higher in the adults), except for fractional area change (FAC). Ejection fraction (EF) and fractional shortening (FS), calculated from M-mode parameters, do not differ between juvenile and adult mice. Stroke volume (SV) and cardiac output (CO) were significantly different between juvenile and adult mice. SV was 31.93 ± 8.67 μl in juveniles vs 70.61 ± 24.66 μl in adults, *ρ* < 0.001. CO was 12.06 ± 4.05 ml/min in juveniles vs 29.71 ± 10.13 ml/min in adults, *ρ* < 0.001. No difference was found in mitral valve (MV) and tricuspid valve (TV) related parameters between juvenile and adult mice. It was demonstrated that variability of M-mode measurements of LV is minimal.

**Conclusions:**

This study suggests that differences in cardiac dimensions, as wells as in pulmonary and aorta outflow parameters, were found between juvenile and adult mice. However, mitral and tricuspid inflow parameters seem to be similar between 3 weeks and 8 weeks mice. The reference values established in this study would contribute as a basis to future studies in post-natal cardiovascular development and diagnosing cardiovascular disorders in genetically modified mouse mutant lines.

## Background

Cardiac ultrasound, also known as echocardiography, is one of the most commonly used diagnostic techniques in human cardiology for possible pathology or lesion. This technique uses high frequency ultrasound waves for visualizing the heart and can provide information on the heart anatomy, blood flow pattern and function of heart muscle, vessels and valves. Until recently, echocardiographic application in animals was limited primarily to larger, non-rodent species. Due to advances in ultrasound imaging technology, ultrasound systems have now the spatial and temporal resolution to obtain accurate and reliable images of mouse hearts [1]. As a result, it has become a valuable non-invasive imaging tool to visualize and evaluate cardiac morphology and function *in vivo* of mice. It was demonstrated by others that echocardiography is becoming a useful technique for studying cardiovascular development and diagnosing cardiovascular disorders in small animals [2].

Knowledge of findings in healthy animals is important for interpretation of results in genetically modified and surgical animals. Most of the available mouse ES cell lines have been generated using the 129/Sv strain. Therefore, the purpose of this study is to provide reference values for transthoracic echocardiography measurements and calculated parameters using B-mode, M-mode and Doppler-mode in juvenile (3 weeks) and adult (8 weeks) 129/Sv mice, obtained during isoflurane anesthesia.

## Methods

### Ethics statement

All animal work performed in this study was conducted compliant with the Portuguese law and approved by the Consultive Commission of the Veterinary Agency (Portuguese Ministry of Agriculture), the sole Agency/Committee in Portugal responsible to issue the ethical approval for these type of studies, following the EU guidelines for animal research and welfare.

### Animals

30 wild type male 129/Sv mice (Harlan Laboratories) divided by two groups, 15 juvenile mice (3 weeks) and 15 adult mice (8 weeks), were studied. The mice were housed in our animal facility in a controlled environment, at 22°C with artificial 12 hours of light/dark cycle, standard diet and free access to water were supplied. The body weight (BW) of each mouse was recorded prior to cardiac examination.

### Echocardiography

Mice were continuously anesthetized by 1.5-2% of isoflurane inhalant mixed with 1 L/min 100% O_2 _to maintain a light sedation level throughout the procedure. They were immobilized on a heating platform ventral side up to maintain the body temperature at 37°C ± 0.5°C. Heart rate (HR) and respiratory physiology were continuously monitored by ECG electrodes. Mice chests were shaved and warmed ultrasound gel was applied to the area of interest. Transthoracic echocardiography was performed using a Vevo 2100 system (VisualSonics, Toronto, Canada) with a 40-MHz transducer. Care was taken to avoid excessive pressure over the sternum, which can distort the signal. Images were captured on cine loops at the time of the study and afterward measurements were done off-line.

### Measurements

The heart was first imaged in B-mode in the parasternal long axis view to examine the left ventricle (LV). The measurements included LV endocardial and epicardial length (LVEndoL and LVEpiL, respectively) in diastole and systole. LV lengths were measured from the aortic annulus to the apex level. For long axis view in B-mode, image depth was 11 mm and image width was 12.08 mm. Moreover, parasternal short axis view was obtained at the level of papillary muscles to measure LV endocardial and epicardial area (LVEndoA and LVEpiA, respectively) in diastole and systole. These measurements were obtained by tracing the endocardial and epicardial border of the LV, where the papillary muscles were excluded from the endocardial tracings. In order to estimate LV mass (LVM) and LV volume (LVV), the area-length method was used [3,4]. LVM was normalized to BW and represented as LVM index (LVMi). Endocardial area change (EAC) and fractional area change (FAC) were also calculated. For short axis view in B-mode, image depth was 10 mm and image width was 9.08 mm.

In order to acquire accurate measurements of cardiac dimensions, M-mode images were obtained from long axis and short axis B-mode images by placing the M-mode sample gate perpendicular to the interventricular septum (IVS) and LV walls, respectively, at the level of papillary muscles. M-mode sample gate depth, length and angle for long axis view were 8–11 mm, 4.6-7.6 mm and 0 dg, respectively. For short axis view, M-mode sample gate depth, length and angle were 8–10 mm, 4.6-7.6 mm and 0 dg, respectively. M-mode from long axis view was performed to measure IVS thickness, while M-mode from short axis view was performed to measure thickness of LV anterior wall (LVAW), LV posterior wall (LVPW) and LV internal diameter (LVID). All M-mode measurements were performed in end-diastole (−d) and end-systole (−s) according to the leading-edge method of the American Society of Echocardiography [5]. End-diastolic and end-systolic measurements were obtained at the time of maximal internal chamber dimensions and at the minimal internal chamber dimensions, respectively [6]. The LV structural parameters measured from short axis view in M-mode were used in the calculation of LV ejection fraction (EF) and LV fractional shortening (FS). M-mode from right parasternal long axis view was performed to evaluate the right ventricle internal diameter (RVID) and thickness of right ventricle anterior wall (RVAW). M-mode sample gate depth, length and angle for the right ventricle (RV) view were 7–9 mm, 2.6-4.6 mm and 0 dg, respectively.

Blood flow was assessed using PW Doppler-mode, by positioning the Doppler sample volume parallel to flow direction, which was assisted by Color Doppler-mode. From a modified short axis view of the pulmonary valve (PV) we measured pulmonary artery diameter (PAD) and PV peak velocity (PVPV). PV peak pressure gradient (PVPPG) was calculated. For pulmonary artery view in B-mode, image depth was 10–11 mm and image width was 9.08 mm. For Doppler mode of pulmonary artery view, Doppler sample volume depth, size and angle were 5–8 mm, 0.22 mm and 5–30 dg, respectively. Ascending aorta valve (AoV) flow was obtained from a suprasternal view to measure AoV peak velocity (AoVPV). The aortic arch view was performed to measure the ascending aorta diameter (AoD) and the descending aorta peak velocity (DAoPV). AoV peak pressure gradient (AoVPPG) was calculated. All arterial diameters were measured in systole, at the time of maximal artery diameter. For aorta view in B-mode, image depth was 8–12 mm and image width was 6–11 mm. Doppler sample volume depth, size and angle for ascending aorta were 6–10 mm, 0.27 mm and 20–55 dg, respectively. For descending aorta, Doppler sample volume depth, size and angle were 6–9 mm, 0.27 mm and 5–30 dg, respectively. Stroke volume (SV) and cardiac output (CO) were calculated using aortic outflow as previously described by others [7]. CO was normalized to BW and represented as CO index (COi). HR was determined from spectral Doppler tracings of the pulmonary artery and ascending aorta flow.

Mitral valve (MV) and tricuspid valve (TV) inflow were assessed from the apical 4 chamber view. The MV measurements performed were the following: MV early wave peak (MVE), MV atrial wave peak (MVA), no flow time (NFT), aortic ejection time (AET), isovolumic relaxation time (IVRT), isovolumic contraction time (IVCT) and MV ejection time (MVET). The MV peak pressure gradient (MVPPG), LV myocardial performance index (LVMPI) and MVE/A ratio were calculated. For mitral valve view, Doppler sample volume depth, size and angle were 7–11 mm, 0.22 mm and 5–30 dg, respectively. TV measurements included TV early wave peak (TVE) and TV atrial wave peak (TVA). The TV peak pressure gradient (TVPPG) and TVE/A ratio were calculated. For tricuspid valve view, Doppler sample volume depth, size and angle were 6–12 mm, 0.29 mm and 5–50 dg, respectively.

All measurements were performed excluding the respiration peaks and obtained in triplicate; the mean or median value was used for data analysis. All calculated parameters were automatically computed by the Vevo 2100 standard measurement package. The equations used by the system are shown in detail (see Additional file 1).

### Intra- and inter-observer variability

The variability of LV M-mode measurements was determined. For intra-observer variability, one examiner analyzed all animals twice, in different occasions. For inter-observer variability, all animals were re-analyzed by a blinded examiner. The percentage of error and the observer variation were calculated. The percentage of error is the difference between two observations divided by the mean and expressed as percentages, while the observer variation is the difference between the two measurements.

### Statistical analysis

Statistical analysis was performed using SPSS software (Version 20). Shapiro-Wilk test was used to assess normality of data. The following parameters were not normally distributed: AoVPV, DAoPV, AoVPPG and RVIDs from juvenile data and LVEndoAs, LVEndoLd and LVEpiLd from adult data. Data are presented as mean ± standard deviation or median with interquartile range (IQR), whenever appropriate. To compare results between the two groups, juvenile and adult mice, Student’s unpaired *t*-test was used for the normally distributed data, while Mann–Whitney U test was used for not normally distributed data. Paired *t*-test was used for within group comparison. Bland-Altman analysis was performed to assess agreement between measurements of intra- and inter-observer measurements variability; in addition Pearson’s correlation was used. For BW and HR association with all the measured parameters, Pearson’s correlation or Spearman’s correlation were used depending if data followed a linear model and if it was normally distributed or not, respectively. A p-value < 0.05 was considered significant.

## Results

Satisfactory images could be captured in all animals. Cardiac examination time was around 30 min, in all animals. All echocardiographic measurements performed in juvenile and adult mice were summarized (Tables 1, 2 and 3).

**Table 1:** Echocardiographic measurements from B-mode images

**Measurement**	**Juvenile**	**n**	**Adult**	**n**	***p***
LVEndoLd (mm)	6.18 (0.57)	15	7.40 (0.58)	15	< 0.001
LVEndoLs (mm)	4.99 ± 0.38	15	6.58 ± 0.45	15	< 0.001
LVEpiLd (mm)	6.38 (0.56)	15	7.65 (0.67)	15	< 0.001
LVEpiLs (mm)	5.35 ± 0.34	15	7.03 ± 0.44	15	< 0.001
LVEndoAd (mm^2^)	6.06 ± 0.87	15	9.87 ± 1.20	15	< 0.001
LVEndoAs (mm^2^)	3.06 (0.98)	15	4.25 (1.12)	15	< 0.001
LVEpiAd (mm^2^)	11.85 ± 1.73	15	19.47 ± 1.71	15	< 0.001
LVEpiAs (mm^2^)	8.84 ± 1.83	15	14.46 ± 1.57	15	< 0.001
EAC (mm^2^)	3.22 ± 0.81	15	5.62 ± 1.16	15	< 0.001
FAC (%)	52.89 ± 10.07	15	56.87 ± 7.80	15	ns
LVVd (μl)	29.57 ± 6.04	15	59.90 ± 8.78	15	< 0.001
LVVs (μl)	11.85 ± 3.19	15	23.32 ± 5.31	15	< 0.001
LVM (mg)	39.68 ± 8.64	15	81.90 ± 9.69	15	< 0.001
LVMi (mg/g)	5.26 ± 1.27	15	4.16 ± 0.42	15	0.005
AoD (mm)	0.93 ± 0.09	15	1.27 ± 0.13	15	< 0.001
PAD (mm)	1.34 ± 0.11	15	1.59 ± 0.13	15	< 0.001

Values are mean ± SD or median (IQR), as appropriate. *p* = statistical significance; ns = not significant. n = 15 for all measurements.

LVEndoL = Left ventricle endocardial length. LVEpiL = Left ventricle epicardial length. LVEndoA = Left ventricle endocardial area. LVEpiA = Left ventricle epicardial area. EAC = Endocardial area change. FAC = Fractional area change. LVV = Left ventricle volume. LVM = Left ventricle mass. LVMi = LVM index. AoD = Ascending aorta diameter. PAD = Pulmonary artery diameter. -d = In diastole. -s = In systole.

**Table 2:** Echocardiographic measurements from M-mode images

**Measurement**	**Juvenile**	**n**	**Adult**	**n**	***p***
LVAWd (mm)	0.61 ± 0.09	15	0.74 ± 0.14	15	0.005
LVAWs (mm)	0.85 ± 0.13	15	1.08 ± 0.18	15	< 0.001
LVIDd (mm)	2.84 ± 0.20	15	3.56 ± 0.19	15	< 0.001
LVIDs (mm)	1.96 ± 0.21	15	2.44 ± 0.28	15	< 0.001
LVPWd (mm)	0.59 ± 0.15	15	0.64 ± 0.13	15	ns
LVPWs (mm)	0.81 ± 0.17	15	0.88 ± 0.19	15	ns
EF (%)	59.63 ± 9.05	15	59.91 ± 8.15	15	ns
FS (%)	30.76 ± 6.15	15	31.45 ± 5.63	15	ns
IVSd (mm)	0.41 ± 0.08	15	0.47 ± 0.15	15	ns
IVSs (mm)	0.50 ± 0.13	15	0.62 ± 0.25	15	ns
RVIDd (mm)	1.08 ± 0.15	7	1.42 ± 0.19	11	0.001
RVIDs (mm)	0.65 (0.31)	7	0.89 (0.21)	11	ns
RVAWd (mm)	0.50 ± 0.11	7	0.32 ± 0.08	11	0.001
RVAWs (mm)	0.66 ± 0.16	7	0.57 ± 0.13	11	ns

Values are mean ± SD or median (IQR), as appropriate. *p* = statistical significance. ns = not significant. n = 15 for all measurements, except for RV measurements (n = 7 for the juveniles and n = 11 for the adults).

LVAW = Left ventricle anterior wall. LVID = Left ventricle internal diameter. LVPW = Left ventricle posterior wall. EF = Ejection fraction. FS = Fractional shortening. IVS = Interventricular septum. RVID = Right ventricle internal diameter. RVAW = Right ventricle anterior wall. -d = In diastole. -s = In systole.

**Table 3:** Echocardiographic measurements from Doppler images

**Measurement**	**Juvenile**	**n**	**Adult**	**n**	***p***
AoVPV (mm/s)	1055.28 (327.21)	15	1557.67 (693.61)	15	0.003
AET (ms)	50.37 ± 4.56	15	49.59 ± 4.50	15	ns
AoVPPG (mmHg)	4.45 (3.13)	15	9.76 (9.46)	15	0.003
DAoPV (mm/s)	747.59 (162.89)	15	1049.88 (321.15)	15	0.002
SV (μl)	31.93 ± 8.67	15	70.61 ± 24.66	15	< 0.001
CO (ml/min)	12.06 ± 4.05	15	29.71 ± 10.13	15	< 0.001
COi (ml/min.g)	1.54 ± 0.36	15	1.48 ± 0.39	15	ns
PVPV (mm/s)	663.34 ± 141.08	15	810.62 ± 149.55	15	0.01
PVPPG (mmHg)	1.83 ± 0.77	15	2.71 ± 0.96	15	0.01
MVE (mm/s)	673.53 ± 149.31	15	747.08 ± 92.31	15	ns
MVA (mm/s)	456.51 ± 73.29	15	500.17 ± 85.14	15	ns
IVCT (ms)	10.90 ± 2.76	15	12.91 ± 3.45	15	ns
IVRT (ms)	16.52 ± 3.29	15	12.48 ± 2.49	15	0.001
MVET (ms)	61.05 ± 9.74	15	59.24 ± 8.34	15	ns
NFT (ms)	77.45 ± 5.66	15	75.66 ± 7.57	15	ns
MVPPG (mmHg)	1.98 ± 0.76	15	2.30 ± 0.52	15	ns
MVE/A	1.50 ± 0.37	15	1.53 ± 0.27	15	ns
LVMPI	0.55 ± 0.14	15	0.51 ± 0.07	15	ns
TVE (mm/s)	241.72 ± 30.57	12	252.83 ± 77.42	6	ns
TVA (mm/s)	400.90 ± 47.96	12	386.53 ± 123.06	6	ns
TVPPG (mmHg)	0.74 ± 0.21	12	0.68 ± 0.39	6	ns
TVE/A	0.60 ± 0.06	12	0.66 ± 0.13	6	ns

Values are mean ± SD or median (IQR), as appropriate. *p* = statistical significance. ns = not significant. n = 15 for all measurements, except for TV measurements (n = 12 for the juveniles and n = 6 for the adults).

AoVPV = Ascending aorta valve peak velocity. AET = Aortic ejection time. AoVPPG = Ascending aorta valve peak pressure gradient. DAoPV = Descending aorta peak velocity. SV = Stroke volume. CO = Cardiac output. COi = CO index. PVPV = Pulmonary valve peak velocity. PVPPG = Pulmonary valve peak pressure gradient. MVE = Mitral valve early wave peak. MVA = Mitral valve atrial wave peak. IVCT = Isovolumic contraction time. IVRT = Isovolumic relaxation time. MVET = Mitral valve ejection time. NFT = No Flow Time. MVPPG = Mitral valve peak pressure gradient. LVMPI = Left ventricle myocardial performance index. TVE = Tricuspid valve early wave peak. TVA = Tricuspid valve atrial wave peak. TVPPG = Tricuspid valve peak pressure gradient.

The RV could only be assessed in 7 juvenile and in 11 adult mice. The TV inflow could only be assessed in 12 juvenile and in 6 adult mice.

BW was significantly lower in the juvenile (7.68 ± 1.33 g) as compared to adult mice (19.84 ± 2.84 g; *p* < 0.001). In the same way, HR was significantly lower in the juvenile (387.34 ± 44.12 bpm) as compared to adult mice (421.82 ± 25.34 bpm; *p* = 0.015).

**Figure 1:**
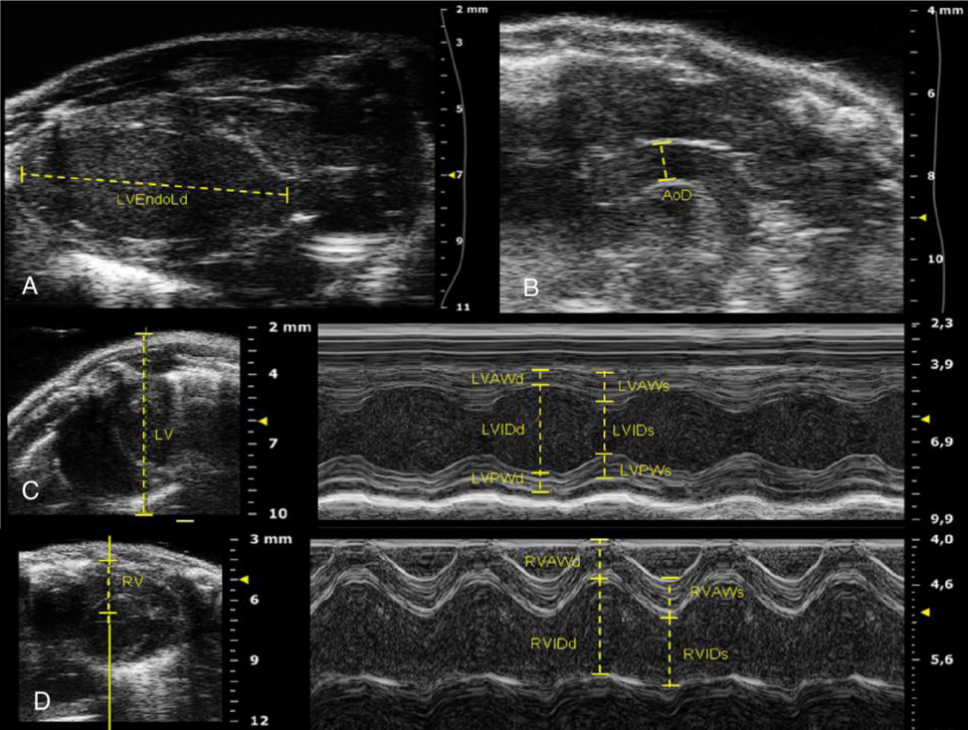
**Representative B-mode and M-mode echocardiographic images and measurements.** (**A**) Parasternal long axis view of left ventricle (LV) in B-mode. (**B**) Aortic arch view in B-mode. (**C**) Short axis view in 2D (left panel) and M-mode tracing (right panel) of the LV, at the level of papillary muscle. (**D**) Right parasternal long axis view in 2D (left panel) and M-mode tracing (right panel) of the righ ventricle (RV). LVEndoL = Left ventricle endocardial length. AoD = Ascending aorta diameter. LVAW = Left ventricle anterior wall. LVID = Left ventricle internal diameter. LVPW = Left ventricle posterior wall. RVAW = Right ventricle anterior wall. RVID = Right ventricle internal diameter; -d = In diastole. -s = In systole.

**Figure 2:**
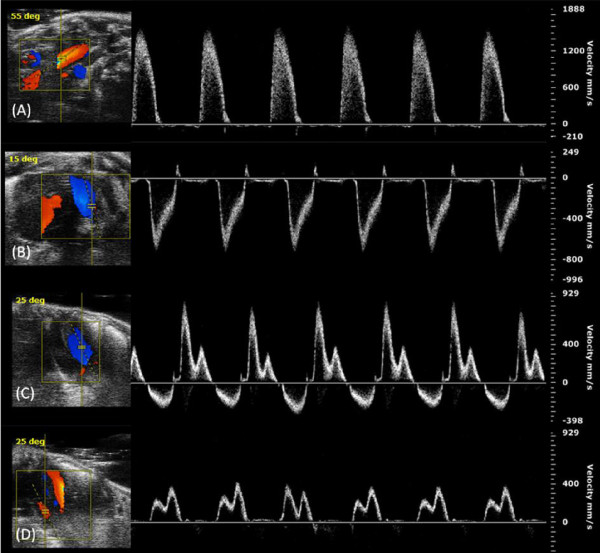
**Representative Doppler echocardiographic images.** (**A**) Suprasternal view (left panel) and PW Doppler tracing (right panel) of aortic outflow. (**B**) Modified short axis view (left panel) and PW-Doppler tracing (right panel) of pulmonary outflow. (**C**) Apical 4 chamber view (left panel) and PW-Doppler tracing (right panel) of mitral valve (MV) inflow. (**D**) Apical 4 chamber view (left panel) and PW-Doppler tracing (right panel) of tricuspide valve (TV) inflow.

**Figure 3:**
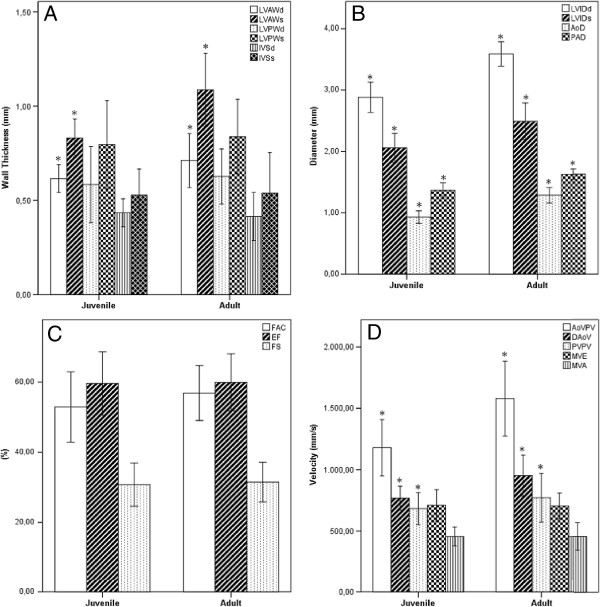
**Bar graphs showing overview over the results of juvenile (n = 15) vs adult mice (n = 15).** (**A**) Thicknesses of left ventricle (LV) walls and interventricular septum (IVS). (**B**) Diameters of LV and arteries. (**C**) Percentages of fractional area change (FAC), ejection fraction (EF) and fractional shortening (FS). (**D**) Peak velocities of arterial outflow and mitral valve (MV) inflow. * Statistically significant. LVID = Left ventricle internal diameter. AoD = Ascending aorta diameter. PAD = Pulmonary artery diameter. LVAW = Left ventricle anterior wall. LVPW = Left ventricle posterior wall. AoVPV = Ascending aorta valve peak velocity. DAoPV = Descending aorta peak velocity. PVPV = Pulmonary valve peak velocity. MVE = Mitral valve early wave peak. MVA = Mitral valve atrial wave peak. -d = In diastole. -s = In systole.

Table 1 summarizes B-mode echocardiographic measurements. This table shows that all parameters have higher values in the adult mice than juvenile, except for FAC and LVMi. Table 2 summarizes M-mode echocardiographic measurements. This table shows that EF and FS are considered the same in the two groups. Representative images and measurements of echocardiographic B-mode and M-mode are shown in Figure 1. Table 3 summarizes Doppler echocardiographic measurements. SV and CO were lower in the juvenile as compared to adult mice. No significant difference was found in MV and TV related data between juvenile and adult mice, except for IVRT. Representative images of echocardiographic PW Doppler-mode are shown in Figure 2. The most important values of Tables 1, 2 and 3 are also summarized in Figure 3.

We also found that some of the structural parameters analyzed, such as AoD, LVEpiLs and LVEndoLd, had a strong positive correlation with BW, both in juvenile and adult mice (see Additional file 2 and Additional file 3). Both CO and SV correlated strongly with BW in juveniles and adults (*p* < 0.01). In 3 weeks mice, NFT had a moderately strong negative correlation with HR (*p* < 0.01) (see Additional file 2). In 8 weeks mice, also NFT and AET had a strong negative correlation with HR (*p* < 0.01) (see Additional file 3).

**Figure 4:**
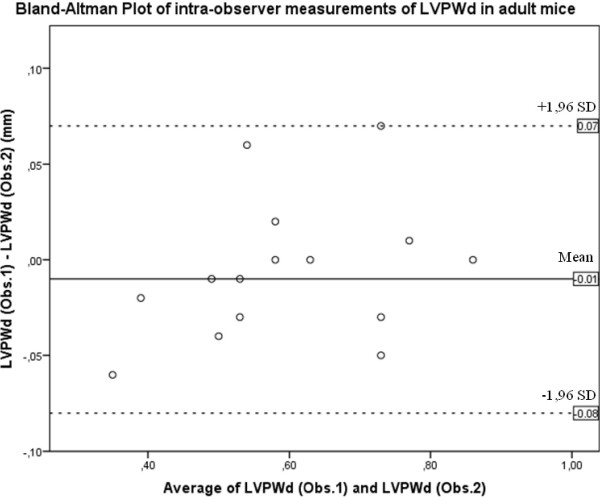
**Bland-Altman correlation of intra-observer measurements of LVPWd in adult mice.** LVPWd = Left ventricle posterior wall in diastole. Obs.1 = Measurement 1 of observer 1. Obs. 2 = Measurement 2 of observer 1.

Table 4 summarizes intra- and inter-observer variability of LV M-mode measurements. No significant differences between measurements were found, except for intra-observer measurement of LVPWd from adult mice (*p* < 0.05 by paired *t*-test). However, Bland-Altman analysis showed a good agreement of both measurements (Figure 4), linear regression LVPWd (intra_Obs.1) = 0.885 × LVPWd (intra_Obs.2) + 0.107, *r*^2^ = 0.89, Pearson’s correlation 0.94, *p* < 0.01. The agreement between intra- and inter-observer measurements was considered high, as illustrated in Bland-Altman analysis (Table 5).

**Table 4:** Variability of LV M-mode measurements

	**Intra-Observer**		**Inter-Observer**	
**Juvenile**	**Error (%)**	**Observer variation (mm)**	**Error (%)**	**Observer variation (mm)**
LVAWd	1.08 ± 6.66	−0.01 ± 0.04	3.05 ± 11.36	0.02 ± 0.06
LVAWs	3.74 ± 5.11	−0.03 ± 0.04	4.88 ± 12.02	0.04 ± 0.10
LVIDd	1.07 ± 3.30	0.03 ± 0.10	0.32 ± 6.29	0.01 ± 0.18
LVIDs	3.11 ± 7.32	0.06 ± 0.16	1.11 ± 14.40	0.02 ± 0.29
LVPWd	1.78 ± 7.11	−0.01 ± 0.04	13.82 ± 27.87	0.07 ± 0.14
LVPWs	0.78 ± 9.52	0.00 ± 0.08	9.15 ± 30.98	0.06 ± 0.20
IVSd	4.96 ± 11.52	0.02 ± 0.04	8.83 ± 24.40	0.05 ± 0.11
IVSs	2.40 ± 13.61	0.00 ± 0.07	2.07 ± 23.71	0.02 ± 0.13
**Adult**	**Error (%)**	**Observer Variation (mm)**	**Error (%)**	**Observer Variation (mm)**
LVAWd	4.18 ± 8.30	−0.03 ± 0.07	3.24 ± 12.83	0.03 ± 0.10
LVAWs	2.40 ± 8.69	−0.02 ± 0.10	1.70 ± 9.05	0.02 ± 0.10
LVIDd	1.49 ± 3.52	0.05 ± 0.12	−0.43 ± 2.77	−0.02 ± 0.10
LVIDs	1.13 ± 5.56	0.03 ± 0.14	0.57 ± 5.44	0.01 ± 0.15
LVPWd	5.68 ± 7.16 *	−0.03 ± 0.04*	−5.64 ± 20.58	−0.05 ± 0.19
LVPWs	2.10 ± 6.29	−0.01 ± 0.05	−8.86 ± 21.86	−0.10 ± 0.24
IVSd	2.73 ± 8.55	−0.01 ± 0.04	−2.88 ± 21.07	−0.01 ± 0.09
IVSs	2.04 ± 10.62	0.00 ± 0.06	−0.84 ± 18.54	0.01 ± 0.10

Values are mean ± SD. *p* = statistical significance. All *p* > 0.05, except * *p* = 0.01.

LVAW = Left ventricle anterior wall. LVID = Left ventricle internal diameter. LVPW = Left ventricle posterior wall. IVS = Interventricular septum. -d = In diastole. -s = In systole.

**Table 5:** Agreement between measurements of intra- and inter-observer variability

	**Intra-Observer**				**Inter-Observer**			
**Juvenile**	**Mean difference ± SD**	**Limits of agreement**	***r***	p*	**Mean difference ± SD**	**Limits of agreement**	***r***	p*
LVAWd	−0.01 ± 0.04	−0.09 to 0.08	0.91	<0.01	0.02 ± 0.06	−0.11 to 0.14	0.82	<0.01
LVAWs	−0.03 ± 0.04	−0.12 to 0.06	0.94	<0.01	0.04 ± 0.10	−0.16 to 0.24	0.72	<0.01
LVIDd	0.03 ± 0.10	−0.17 to 0.23	0.89	<0.01	0.01 ± 0.18	−0.36 to 0.38	0.64	<0.05
LVIDs	0.06 ± 0.16	−0.25 to 0.38	0.77	<0.01	0.02 ± 0.29	−0.55 to 0.60	0.20	ns
LVPWd	−0.01 ± 0.04	−0.08 to 0.07	0.97	<0.01	0.07 ± 0.14	−0.21 to 0.36	0.50	ns
LVPWs	0.00 ± 0.08	−0.16 to 0.15	0.89	<0.01	0.06 ± 0.20	−0.34 to 0.46	0.42	ns
IVSd	0.02 ± 0.04	−0.07 to 0.11	0.83	<0.01	0.05 ± 0.11	−0.17 to 0.26	0.39	ns
IVSs	0.00 ± 0.07	−0.13 to 0.14	0.91	<0.01	0.02 ± 0.13	−0.24 to 0.27	0.57	<0.05
**Adult**	**Mean difference ± SD**	**Limits of agreement**	***r***	p*	**Mean difference ± SD**	**Limits of agreement**	***r***	p*
LVAWd	−0.03 ± 0.07	−0.17 to 0.10	0.90	<0.01	0.03 ± 0.10	−0.18 to 0.23	0.70	<0.01
LVAWs	−0.02 ± 0.10	−0.22 to 0.17	0.84	<0.01	0.02 ± 0.10	−0.19 to 0.23	0.82	<0.01
LVIDd	0.05 ± 0.12	−0.18 to 0.28	0.87	<0.01	−0.02 ± 0.10	−0.22 to 0.18	0.91	<0.01
LVIDs	0.03 ± 0.14	−0.24 to 0.30	0.89	<0.01	0.01 ± 0.15	−0.29 to 0.30	0.91	<0.01
LVPWd	−0.03 ± 0.04	−0.12 to 0.05	0.94	<0.01	−0.05 ± 0.19	−0.42 to 0.32	0.47	ns
LVPWs	−0.01 ± 0.05	−0.12 to 0.10	0.96	<0.01	−0.10 ± 0.24	−0.57 to 0.38	0.49	ns
IVSd	−0.01 ± 0.04	−0.08 to 0.06	0.97	<0.01	−0.01 ± 0.09	−0.18 to 0.17	0.83	<0.01
IVSs	0.00 ± 0.06	−0.11 to 0.11	0.98	<0.01	0.01 ± 0.10	−0.18 to 0.21	0.93	<0.01

*r* = Pearson’s coefficient. *p-value for correlation coefficient.

LVAW = Left ventricle anterior wall. LVID = Left ventricle internal diameter. LVPW = Left ventricle posterior wall. IVS = Interventricular septum. -d = In diastole. -s = In systole.

## Discussion

In order to understand and analyze studies that use transgenic animals or animals that undergo surgical procedures, the cardiac characterization of normal/wild type and healthy animals is considered extremely important. To our best knowledge, echocardiographic evaluation of reference values of B-mode, M-mode and Doppler-mode in juvenile (3 weeks) and adult (8 weeks) 129/Sv wild-type mice has not been reported. In the present study, cardiac dimensions were significantly different between juvenile and adult mice, as expected. Diastolic function does not differ between juvenile and adult mice. Additionally, we demonstrate that the variability of LV measurements in M-mode is minimal, indicating that this method is reliable.

### Body weight and heart rate

The BW was significantly lower in the juvenile than in the adult mice, as expected. Similar results were observed in other mice strains, as C57BL/6 and CD1 mice [6,8,10]. Nevertheless, BW of 3 weeks 129/Sv mice was lower than 3 weeks C57BL/6 mice (7.68g vs 10.2g) [10] and 8 weeks 129/Sv mice were lower than 8 weeks CD1 mice (19.84g vs 32.4g) [6], showing the influence of mouse backgrounds in body weight. In addition, HR was significantly lower in the younger animals, despite the similar isoflurane concentration used for both groups, which is inconsistent with some studies [6,8,11]. One study shows that HR in C57BL6 mice is constant between 1 month and 2 months mice and decreases between 2 months and 16 months [8], while other studies show that HR in C57BL6 conscious mice and CD1 mice decreases with age [6,11]. We found one study in accordance with our result, where HR was higher in old vs young C57BL/6 mice [10]. The higher HR observed in the adult mice might be explained possibly due to a higher thoracic compression in the adult animals during echocardiography, to allow a better access to the heart.

### Cardiac dimensions parameters

We observed a significant difference in LV lengths and LV areas in 3 weeks mice vs 8 weeks mice, which could be explained by the significantly higher BW of adult mice, due to the continuous increase of the heart and body weight between these ages.

A higher value of EAC was observed in the adult mice, but we couldn’t find any data to correlate with our result. While FAC, a parameter that represents LV systolic function, does not differ between juvenile and adult. According to previous studies in C57BL6 mice, FAC does not differ between these ages, which correlate with our result. However the value found is lower than in our study, around 46% [8]. Together, these results reinforce the influence of mice background on cardiac parameters.

As expected, LVV and LVM were different between 3 weeks mice and 8 weeks mice, since the animals are still in growth. However, when LVM was normalized to BW (LVMi), we noticed that LVMi was higher in the juveniles due to the higher BW of the adult mice. These results are consistent with the literature found for other strains [6,12].

We observed no significant difference in thickness of IVS and LVPW between juvenile and adult mice. A similar result was obtained in a previous study with other mice strain between 6 and 12 weeks old [9]. LVAW thickness and LVID were significantly different in the two groups. The literature found for these parameters showed a tendency toward an increase of LVAW [6] and LVID [8,9] with age, although no statistical significance was reached.

Our data suggests that EF and FS, parameters of systolic function, does not differ between 3 weeks and 8 weeks mice, which match with previous results found for other mice strains [6,8,9]. The values found for EF in other studies with anesthetized animals were around 53 – 55% for 129/Sv mice [13] and around 60 – 63% for CD1 mice [6]. EF in conscious animals was around 65% for C57BL6 mice [14] and 84 % for 129/Sv mice [13]. The values found for FS in other studies with anesthetized animals were around 37 – 40% for CD1 mice [6], around 33 – 35% for 129/Sv mice [13] and around 35% for C57BL6 mice [8]. In another study, a FS value of 45% was observed for 6–8 months C57BL6 mice [15]. FS in conscious animals were around 51% for 129/Sv mice [13].

### Outflow-related parameters

Concerning the aorta and pulmonary artery, their respective diameters, peak velocities and peak pressures were different between juvenile and adult mice. The higher arterial diameters in the adults, due to higher body surface in older animals, go along with the literature found [8]. AET was the only arterial-related parameter that did not show a significant difference between the two groups.

SV and CO, calculated by the aortic outflow method, were significantly higher in 8 weeks mice. On the other hand, COi was considered the same in the two groups due to BW normalization of CO resulted from higher BW in the adult mice compared to lower BW in the juvenile mice. Parameters that are constant when indexed to BW indicate that these values are related to body size. Our CO result is inconsistent with the data found for other strain [6], which shows no difference between different ages. This could be explained by the older ages tested in the referred study (between 8 and 52 weeks). The values found in other studies for CO were around 18 – 20 ml/min for 129/Sv mice [13] and 16 – 17 ml/min for CD1 mice [6].

### Inflow-related parameters

We did not find any significant difference in MV and TV inflow parameters between the two groups, except for IVRT that was significantly lower in the 8 weeks mice. E wave and A wave are diastolic parameters that depend mainly on myocardial relaxation, LV geometry and loading conditions. Therefore during maturation of the LV, from juvenile to adult, these parameters evolve in parallel, keeping the resulting E and A wave constant. IVRT is dependent of LV relaxation, loading conditions and HR. Bearing in mind the constant values of A and E wave, IVRT changes could be dependent on intrinsic myocardial relaxation. A similar result was obtained for MVE [6,8], MVA [6], MVPPG [6], IVRT [8] and MVE/A [6,8] for other mice strains in previous studies.

### Limitations

One limitation of this study is the influence of anesthetic agent as depressor of cardiovascular function. However the use of anesthesia during echocardiography is crucial to facilitate data acquisition, by providing sedation and immobility of animals, avoiding the animal stress during the procedure. We used the isoflurane, since it’s one of the most common inhalant anesthetics, and has many advantages when compared with other anesthetics. It produces minimal cardiac depression and has higher molecular stability [16]. Also, isoflurane was considered the most reproducible anesthetic in repeat studies at 12 days [17].

It would have been advantageous to consider more than two time points, in order to analyze data during the entire life span of these animals.

Also, it would be interesting to analyze, in the future, global and regional strain data for deformations reference values.

## Conclusions

Since mice are a common model system for studying cardiac development and disease, cardiac characterization of normal/wild type and healthy animals is considered extremely important for interpretation of results in transgenic and surgical animals. And so, this study defines reference echocardiographic measurements and calculated parameters for juvenile (3 weeks) and adult (8 weeks) 129/Sv wild type mice.

The work presented here suggests a significant difference of almost all cardiac dimensions as well as outflow-related parameters (pulmonary and aorta flow) of 3 weeks vs 8 weeks 129/Sv wild type mice. Interestingly, inflow-related parameters (mitral and tricuspid flow) do not differ between weeks 3 and 8. In addition, when comparing with the literature, we can detect some differences in the parameters studied, suggesting the great influence of genetic background of each mice strain on the results obtained.

The reference values reported in this study could contribute as a basis to further comparative results between strains and to future studies in cardiovascular postnatal development and diagnosing cardiovascular disorders using murine models in the 129Sv genetic background.

## Competing interests

The authors declare that they have no competing interests.

## Authors’ contributions

MV performed the image acquisitions and measurements, performed statistical analysis, analyzed and interpreted the ultrasound data and wrote the manuscript. ACA performed the measurements for the inter-observer variability, participated in the interpretation of the results and contributed to the writing of the manuscript. SR participated in the interpretation of the results and contributed to the writing of the manuscript. LBR participated in the interpretation of the results and contributed to the writing of the manuscript. JAB designed the study and wrote the manuscript. All authors have read and approved the final manuscript.

## Supplementary Material

Additional file 1 Equations automatically computed by the Vevo 2100 system. Table showing equations automatically computed by the Vevo 2100 system. Click here for file

Additional file 2 **Correlation analysis between BW or HR and all echocardiographic parameters measured in juvenile mice.** Table showing correlation analysis between BW or HR and all echocardiographic parameters measured in juvenile mice. Click here for file

Additional file 3 **Correlation analysis between BW or HR and all echocardiographic parameters measured in adult mice.** Table showing correlation analysis between BW or HR and all echocardiographic parameters measured in adult mice. Click here for file
