# Supplementary material for: Transthoracic echocardiography reference values in juvenile and adult 129/Sv mice
Source: Cardiovasc Ultrasound. 2013 May 1;11:12. doi: 10.1186/1476-7120-11-12 (PMC3651272; doi:10.1186/1476-7120-11-12)
Supplement: Additional file 1 — Equations automatically computed by the Vevo 2100 system. Table showing equations automatically computed by the Vevo 2100 system. [file 1476-7120-11-12-S1.docx]

**Additional file 1.** **Equations automatically computed by the Vevo 2100 system.**

| **Description** | **Equation** |
| --- | --- |
| Left ventricle volume in diastole (µl) | $LVVd=\frac{3}{4}\times LVEndoLd\times LVEndoAd$ |
| Left ventricle volume in systole (µl) | $LVVs=\frac{3}{4}\times LVEndoLs\times LVEndoAs$ |
| Left ventricle mass (mg) | $LVM=1.05\times\left( \frac{5}{6} \times LVEpiAd\times\left( LVEpiLd+Td \right) \right)-\left( \frac{5}{6}\times LVEndoAd\times LVEndoLd \right)$ |
| Endocardial area change (mm^2^) | $EAC=LVEndoAd-LVEndoAs$ |
| Fractional area change (%) | $FAC=\frac{LVEndoAd-LVEndoAs}{\mathrm{LVEndoAd}}\times100$ |
| Ejection fraction (%) | $EF=100\times\left( \frac{LVVd-LVVs}{\mathrm{LVVd}} \right)$ |
| Fractional shortening (%) | $FS=100\times\left( \frac{LVIDd-LVIDs}{\mathrm{LVIDd}} \right)$ |
| Stroke Volume (µl) | $SV=0.785\times\left( \mathrm{AoD} \right)^{2}\times AoVTI$ |
| Cardiac output (ml/min) | $CO=\frac{SV\times HR (from AoD)}{1000}$ |
| Aortic valve peak pressure gradient (mmHg) | $AoVPPG=4\times\left( \frac{\mathrm{AoVPV}}{1000} \right)^{2}$ |
| Pulmonary valve peak pressure gradient (mmHg) | $PVPPG=4\times\left( \frac{\mathrm{PVPV}}{1000} \right)^{2}$ |
| Mitral valve peak pressure gradient (mmHg) | $MVPPG=4\times\left( \frac{\mathrm{MVPV}}{1000} \right)^{2}$ |
| Left ventricle myocardial performance index | $LVMPI=\frac{IVRT+IVCT}{\mathrm{AET}}$ |
| Tricuspid valve peak pressure gradient (mmHg) | $TVPPG=4\times\left( \frac{\mathrm{TVPV}}{1000} \right)^{2}$ |

LVEndoL = Left ventricle endocardial length. LVEndoA = Left ventricle endocardial area. LVEpiA = Left ventricle epicardial area. LVEpiL = Left ventricle epicardial length. T = Average wall thickness. LVV = Left ventricle volume. LVID = Left ventricle internal diameter. AoD = Ascending aorta diameter. AoVTI = Ascending aorta velocity time integral. SV = Stroke volume. HR = Heart rate. AoVPV = Ascending aorta valve peak velocity. PVPV = Pulmonary valve peak velocity. MVPV = Mitral valve peak velocity. IVRT = Isovolumic relaxation time. IVCT = Isovolumic contraction time. AET = Aortic ejection time. TVPV = Tricuspid valve peak velocity. -d = In diastole. -s = In systole.
