# Supplementary material for: Transthoracic echocardiography reference values in juvenile and adult 129/Sv mice
Source: Cardiovasc Ultrasound. 2013 May 1;11:12. doi: 10.1186/1476-7120-11-12 (PMC3651272; doi:10.1186/1476-7120-11-12)
Supplement: Additional file 3 — Correlation analysis between BW or HR and all echocardiographic parameters measured in adult mice. Table showing correlation analysis between BW or HR and all echocardiographic parameters measured in adult mice. [file 1476-7120-11-12-S3.docx]

**Additional file 3.** C**orrelation analysis between BW or HR and all echocardiographic parameters measured in adult mice.**

|  |  | | **Adult Mice (8 Weeks)** | | |  | |  | |
| --- | --- | --- | --- | --- | --- | --- | --- | --- | --- |
|  | **BW** | | **HR** | |  | **BW** | | **HR** | |
| **Parameter** | **Correlation Coefficient** | ***p*** | **Correlation Coefficient** | ***p*** | **Parameter** | **Correlation Coefficient** | ***p*** | **Correlation Coefficient** | ***p*** |
| AoD | 0.75* | 0.001 | 0.23 | ns | IVSd | -0.30 | ns | -0.14 | ns |
| PAD | 0.30 | ns | 0.35 | ns | IVSs | -0.07 | ns | 0.03 | ns |
| CO | 0.67 | < 0.01 | 0.22 | ns | RVIDd (n=11) | 0.56 | ns | 0.48 | ns |
| SV | 0.73 | < 0.01 | 0.05 | ns | RVIDs (n=11) | 0.31 | ns | -0.09 | ns |
| LVEndoLd | 0.81 | 0 | -0.01 | ns | RVAWd (n=11) | 0.21 | ns | -0.40 | ns |
| LVEndoLs | 0.71* | < 0.01 | -0.14 | ns | RVAWs (n=11) | 0.39 | ns | -0.06 | ns |
| LVEpiLd | 0.79 | 0.001 | 0.01 | ns | AoVPV | 0.58 | < 0.05 | -0.02 | ns |
| LVEpiLs | 0.72* | < 0.01 | -0.09 | ns | AET | -0.05 | ns | -0.75 | 0.001 |
| LVEndoAd | 0.33 | ns | 0.38 | ns | AoVPPG | 0.58 | < 0.05 | -0.02 | ns |
| LVEndoAs | 0.15 | ns | 0.02 | ns | DAoPV | -0.09 | ns | 0.24 | ns |
| LVEpiAd | 0.51 | ns | 0.27 | ns | PVPV | -0.07 | ns | 0.38 | ns |
| LVEpiAs | 0.24 | ns | 0.07 | ns | PVPPG | -0.07 | ns | 0.38 | ns |
| EAC | 0.30 | ns | 0.42 | ns | MV E | -0.29 | ns | 0.04 | ns |
| FAC | 0.07 | ns | 0.24 | ns | MV A | -0.11 | ns | 0.52 | < 0.05 |
| LVVd | 0.40 | ns | 0.34 | ns | MVPPG | -0.37 | ns | 0.05 | ns |
| LVVs | 0.24 | ns | 0.03 | ns | IVCT | 0.34 | ns | -0.37 | ns |
| LVM | 0.71* | < 0.01 | 0.10 | ns | IVRT | 0.40 | ns | -0.31 | ns |
| LVAWd | 0.20 | ns | 0.16 | ns | MV ET | 0.10 | ns | -0.53 | < 0.05 |
| LVAWs | 0.23 | ns | 0.16 | ns | NFT | 0.20 | ns | -0.64 | 0.01 |
| LVIDd | 0.23 | ns | 0.24 | ns | MV E/A | -0.17 | ns | -0.22 | ns |
| LVIDs | 0.01 | ns | -0.08 | ns | LV MPI | 0.64 | 0.01 | -0.10 | ns |
| LVPWd | 0.49 | ns | -0.16 | ns | TV E (n=6) | -0.43 | ns | -0.03 | ns |
| LVPWs | 0.19 | ns | 0.12 | ns | TV A (n=6) | -0.14 | ns | 0.09 | ns |
| EF | 0.11 | ns | 0.28 | ns | TVPPG (n=6) | -0.14 | ns | 0.09 | ns |
| FS | 0.11 | ns | 0.28 | ns | TV E/A (n=6) | -0.82* | < 0.05 | 0.75 | ns |

For all parameters correlation coefficient are from Spearman’s correlation, except *. *Pearson’s correlation coefficient. *p* = statistical significance. ns = not significant. n=15 for all measurements, except for RV measurements (n=11 for the adult mice) and for TV measurements (n=6 for the adult mice).

BW = Body weight. HR = Heart rate. AoD = Ascending aorta diameter. PAD = Pulmonary artery diameter. CO = Cardiac output. SV = Stroke volume. LVEndoL = Left ventricle endocardial length. LVEpiL = Left ventricle epicardial length. LVEndoA = Left ventricle endocardial area. LVEpiA = Left ventricle epicardial area. EAC = Endocardial area change. FAC = Fractional area change. LVV = Left ventricle volume. LVM = Left ventricle mass. LVAW = Left ventricle anterior wall. LVID = Left ventricle internal diameter. LVPW = Left ventricle posterior wall. EF = Ejection fraction. FS = Fractional shortening. IVS = Interventricular septum. RVID = Right ventricle internal diameter. RVAW = Right ventricle anterior wall. AoVPV = Ascending aorta valve peak velocity. AET = Aortic ejection time. AoVPPG = Ascending aorta valve peak pressure gradient. DAoPV = Descending aorta peak velocity. PVPV = Pulmonary valve peak velocity. PVPPG = Pulmonary valve peak pressure gradient. MVE = Mitral valve early wave peak. MVA = Mitral valve atrial wave peak. MVPPG = Mitral valve peak pressure gradient. IVCT = Isovolumic contraction time. IVRT = Isovolumic relaxation time. MVET = Mitral valve ejection time. NFT = Non-filling time. LVMPI = Left ventricle myocardial performance index. TVE = Tricuspid valve early wave peak. TVA = Tricuspid valve atrial wave peak. TVPPG = Tricuspid valve peak pressure gradient. -d = In diastole. -s = In systole.
